# Epilepsy in adults with mitochondrial disease: A cohort study

**DOI:** 10.1002/ana.24525

**Published:** 2015-11-17

**Authors:** Roger G. Whittaker, Helen E. Devine, Grainne S. Gorman, Andrew M. Schaefer, Rita Horvath, Yi Ng, Victoria Nesbitt, Nichola Z. Lax, Robert McFarland, Mark O. Cunningham, Robert W. Taylor, Douglass M. Turnbull

**Affiliations:** ^1^ Institute of Neuroscience, Henry Wellcome Building for Neuroecology, Newcastle UniversityNewcastle upon TyneUnited Kingdom; ^2^ Wellcome Trust Center for Mitochondrial Research, Institute of Neuroscience, Newcastle UniversityNewcastle upon TyneUnited Kingdom; ^3^ Institute of Genetic Medicine, International Center for Life, Newcastle UniversityNewcastle upon TyneUnited Kingdom

## Abstract

**Objective:**

The aim of this work was to determine the prevalence and progression of epilepsy in adult patients with mitochondrial disease.

**Methods:**

We prospectively recruited a cohort of 182 consecutive adult patients attending a specialized mitochondrial disease clinic in Newcastle upon Tyne between January 1, 2005 and January 1, 2008. We then followed this cohort over a 7‐year period, recording primary outcome measures of occurrence of first seizure, status epilepticus, stroke‐like episode, and death.

**Results:**

Overall prevalence of epilepsy in the cohort was 23.1%. Mean age of epilepsy onset was 29.4 years. Prevalence varied widely between genotypes, with several genotypes having no cases of epilepsy, a prevalence of 34.9% in the most common genotype (m.3243A>G mutation), and 92.3% in the m.8344A>G mutation. Among the cohort as a whole, focal seizures, with or without progression to bilateral convulsive seizures, was the most common seizure type. Conversely, all of the patients with the m.8344A>G mutation and epilepsy experienced myoclonic seizures. Patients with the m.3243A>G mutation remain at high risk of developing stroke‐like episodes (1.16% per year). However, although the standardized mortality ratio for the entire cohort was high (2.86), this ratio did not differ significantly between patients with epilepsy (2.96) and those without (2.83).

**Interpretation:**

Epilepsy is a common manifestation of mitochondrial disease. It develops early in the disease and, in the case of the m.3243A>G mutation, often presents in the context of a stroke‐like episode or status epilepticus. However, epilepsy does not itself appear to contribute to the increased mortality in mitochondrial disease. Ann Neurol 2015;78:949–957

Mitochondrial diseases are a diverse group of multisystem disorders caused by mutations of mitochondrial DNA (mtDNA).^1^, ^2^ Mitochondria are the main source of cellular adenosine triphosphate through the process of oxidative phosphorylation, and common to all mitochondrial disease is the vulnerability of highly energy‐dependent tissues such as skeletal muscle and brain.^3^ There are many different genetic defects of mtDNA, including both point mutations and large‐scale rearrangements and deletions. In general, a given genetic defect may cause a specific clinical syndrome, but the relationship between genotype and phenotype is not absolute and there is considerable overlap between syndromes.^4^ Nevertheless, some clinical features are more common in certain genotypes. Thus, for example, central nervous system (CNS) involvement is common in the m.3243A>G and m.8344A>G point mutations,^5^ but relatively rare in patients with single mtDNA deletions.^6^

In adults with mitochondrial disease, CNS involvement includes progressive cognitive decline,^7^ migraine,^8^ and epilepsy. Some genotypes produce relatively specific CNS syndromes. For example, in the syndrome of *M*itochondrial *E*ncephalomyopathy, *L*actic *A*cidosis and *S*troke‐Like Episodes, most commonly resulting from the m.3243A>G point mutation,^9^, ^10^ patients suffer repeated episodes of encephalopathy and stroke‐like episodes, usually accompanied by focal seizures in the parietal and occipital lobes. Focal and generalized status epilepticus can occur in association with these stroke‐like episodes. In other syndromes, for example, *M*yoclonic *E*pilepsy with *R*agged *R*ed *F*ibers, usually as a result of the m.8344A>G point mutation, epilepsy is one of the defining clinical features and typically manifests as generalized myoclonus with photosensitivity.^11^, ^12^, ^13^, ^14^ As with other clinical features, the type of epilepsy found only partially reflects the underlying genotype, with a large degree of overlap found between syndromes.^15^, ^16^

Although there have been many reports of epilepsy in mitochondrial disease, these have tended to be in either pediatric series and/or of small numbers of adult patients with sometimes poorly defined molecular defects.^17^, ^18^, ^19^ Furthermore, the prevalence rates for epilepsy vary widely between reports.^20^, ^21^ In this article, we present the first large‐scale cross‐sectional cohort study of the prevalence and classification of epilepsy in genetically defined adult patients with mitochondrial disease and define, for the first time, the epilepsy‐related morbidity and mortality in these patients.

## Subjects/Materials and Methods

All consecutive adult patients (defined as age 16 years or over) attending a specialist mitochondrial clinic between May 1, 2005 and May 1, 2008 were included in the study. Our center in Newcastle upon Tyne provides one of three nationally commissioned specialist mitochondrial disease clinics and receives referrals from throughout the UK and Ireland. One hundred eighty‐two different patients (98 females, 84 males) were observed during this period. Mean age at recruitment to the cohort was 37.8 years (range, 16–58). All patients had proven mitochondrial disease on the basis of molecular genetic, biochemical, and histochemical analysis and were classified on the basis of their genetic diagnosis. Patients were screened by a consultant neurologist using the Newcastle Mitochondrial Disease Adult Scale, a validated rating scale that includes a question on current or past history of epileptic seizures.^22^ Patients with a history of epilepsy were then further interviewed either in clinic or over the telephone to determine seizure frequency and type according to the 1989 International League Against Epilepsy (ILAE) classification.^23^ These were subsequently reclassified according to the 2010 revised ILAE guidelines.^24^ Epilepsy was defined as any history of more than one unprovoked seizure or a period of status epilepticus. Active epilepsy was defined either as one or more seizures within the previous 12 months and a history of previous seizures, two or more seizures in the preceding 12‐month period with no history of previous seizures, or a period of status epilepticus with or without a previous history of epilepsy. Where patients had more than one seizure type, these were classified as primary, secondary, or tertiary based on seizure frequency rather than order of presentation. The hospital notes of all patients were also reviewed in case any patient was not aware of having had previous seizures, and to identify causes of epilepsy other than mitochondrial disease.

The electroencephalograms (EEGs) of all patients were reviewed by a consultant clinical neurophysiologist (R.G.W.), and in patients who had a previous EEG this was not repeated unless clinically indicated. Where patients had undergone several EEGs, the most recent was reviewed. Abnormalities were scored according to the criteria used in the SANAD study.^25^

Having defined our initial cohort, we then followed up these patients through their routine clinic appointments. These typically occurred at intervals of 6 or 12 months. Patients were asked at each visit about the occurrence of seizures or stroke‐like episodes. A further review of the hospital notes was performed in 2015 to ensure that no history of epilepsy had been missed. Outcome measures were the development of epilepsy in those initially seizure free, the occurrence of significant seizure‐related morbidities (ie, stroke‐like episodes or status epilepticus), and death. For all outcome measures, person time at risk was calculated for each individual patient from the year of recruitment to the study. Standardized mortality ratios were calculated using data from the UK Office for National Statistics mortality data, 2010. These data are subdivided into <15, 15 to 34, 35 to 59, 60 to 79, and >80 years age ranges, and the age range within which the mean age of each subgroup fell was used as the denominator in calculations of standardized mortality ratios. The study was approved by Newcastle and North Tyneside Local Research Ethics Committee.

## Results

### Point Prevalence and Phenotype of Epilepsy

**Figure 1:**
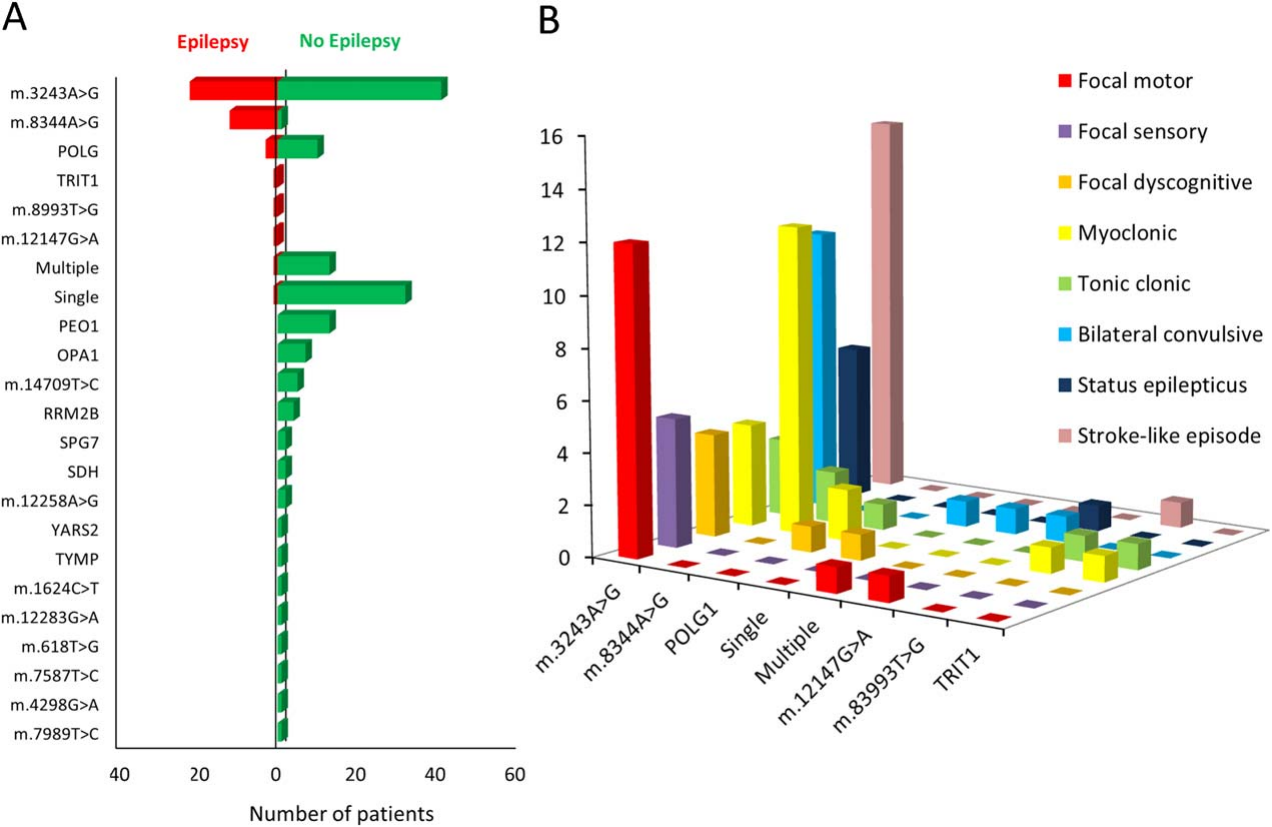
(A) Prevalence of epilepsy among a cohort of 182 adult patients with mitochondrial disease. Overall prevalence of epilepsy was 23.1%. The genotypes most commonly associated with epilepsy were m.3243A>G, m.8344A>G, and recessive *POLG* mutations and represented 11.8%, 6.4%, and 1.6% of the total cohort, respectively. One patient each with the m.12147A>G mutation, m.8993T>G mutation, single large‐scale deletion, multiple mtDNA deletions, and recessive *TRIT1* mutations had epilepsy; together, this group represented 2.7% of the total cohort. (B) Seizure phenotypes for the eight genotypes in which epilepsy occurred. The total number of seizure types is greater than the number of patients with epilepsy because several patients had more than one seizure type. mtDNA = mitochondrial DNA.

One hundred eighty‐two different patients were seen between May 1, 2005 and May 1, 2008. Forty‐two of these had a history of epilepsy, giving a combined prevalence of epilepsy for all genotypes of 23.1% on May 1, 2008 (Figure 1, Table 1). Thirty‐five patients (19.2%) had active epilepsy, whereas the remaining 7 (3.8%) had a previous history of epilepsy but with no seizures in the 12‐month period preceding inclusion. Mean age of onset was 29.4 years (range, 2–58). Epilepsy was a feature of the eight different genotypes of mitochondrial disease listed below.

#### m.3243A>G Mutation

Sixty‐three patients with the m.3243A>G point mutation were included. This group included asymptomatic carriers, patients with isolated diabetes and deafness, and patients with MELAS syndrome. Twenty‐two of the patients with the m.3243A>G point mutation (34.9%) had epilepsy. These patients represented 12.1% of the entire cohort. Mean age of epilepsy onset was 32.6 years (range, 9–56). Five of the 22 patients with a history of epilepsy (22.7%) were seizure free at the time of assessment. In the patients with epilepsy, the most common seizure phenotype was focal motor seizures (12 of 22 = 54.5%). Ten patients (45.5%) had focal seizures evolving to bilateral convulsive seizures. Focal sensory seizures were noted in 5 (22.7%) and focal dyscognitive, or myoclonic, seizures were each noted in 4 (18.2%) of the patients. Three patients (13.6%) had tonic‐clonic seizures.

Eighteen patients had had an EEG; in 4 patients, this was normal, and 14 showed a slowing in background brain activity. Focal, multifocal, and generalized epileptiform abnormalities were noted in 4, 4, and 3 patients, respectively.

#### m.8344A>G Mutation

Thirteen patients with the m.8344A>G point mutation were included. Twelve of these 13 (92.3%) patients had epilepsy, and all of these had myoclonic seizures. These patients represented 6.6% of the entire cohort. The mean age of epilepsy onset was 23.8 years (range, 18–58). In many patients, the myoclonic jerks were subtle and were only revealed on direct questioning. Two patients (16.7%) also had infrequent (<1/month) tonic‐clonic seizures in response to photic stimulation, for example, strobe lighting in nightclubs, or when a passenger in a car. Because these patients had learned to avoid these precipitants, their tonic‐clonic seizures occurred less than once a month. Despite experiencing daily myoclonic jerks, 3 of these patients were not taking antiepileptic medication.

Ten patients had EEGs performed; in 8 of these, the background was slow, and generalized epileptiform abnormalities were noted in 6 patients. Despite the clinical history of photosensitivity in 4 patients, only 1 showed a photoparoxysmal response on their EEG.

#### *Recessive* POLG *Mutations*

Thirteen patients were included, of whom 3 (23.1%) had epilepsy. These patients represented 1.6% of the entire cohort. Mean age of epilepsy onset was 27.5 years (range, 26–33). One patient (biallelic p.(Trp748Ser) and p.(Arg1096Cys) variants) had both myoclonic and tonic‐clonic seizures on a daily basis. An EEG at age 55 showed a slow background with multifocal epileptiform abnormalities. The second patient (recessive p.(Ala467Thr) and p.(X1240Q) had infrequent (<1/month) focal dyscognitive seizures. An EEG at age 42 showed a slow background with no epileptiform abnormalities. The third patient (p.(Ala467Thr) and p.(Arg1096Cys) variants) had previously had myoclonic seizures, but was currently seizure free and on no medication. An EEG at age 45 showed a slow background with no epileptiform abnormalities.

#### Single Deletion

Thirty‐three patients with a single, large‐scale mtDNA deletion were included. Only 1 of these patients had epilepsy (3.0%) with focal dyscognitive seizures occasionally evolving to bilateral convulsive seizures occurring less than once a month. The patient had presented at the age of 5 years with seizures, for which no cause was detected. It was more than 20 years later that the patient presented with ptosis and a diagnosis of mitochondrial disease was made. No EEGs were available from his original presentation with epilepsy and this was not repeated.

#### Multiple mtDNA Deletions

Fourteen patients with multiple mtDNA deletions for which no nuclear gene defect of mtDNA maintenance could be determined were included. One of these 14 patients (7.1%) had epilepsy. This patient presented at the age of 2 years with a mixture of focal motor and bilateral convulsive seizures. She had remained seizure free on two antiepileptic drugs until the age of 59, when she represented with a further flurry of focal motor seizures. She had a normal EEG age 59.

#### m.12147G>A Mutation

Only 1 patient with this rare mtDNA genotype was included. This patient presented at age 17 with a devastating MELAS‐like phenotype with encephalopathy, stroke‐like episode, and focal status epilepticus affecting the right temporal lobe. The degree of edema after the stroke‐like episode was so great that a temporal lobectomy was performed in order to reduce intracranial pressure. At the time of assessment at age 26, the patient was seizure free on carbamazepine. An EEG performed at the age of 23 showed a slow background with focal epileptiform abnormalities over the temporal region.

#### m.8993T>G Mutation

One patient with the m.8993T>G point mutation was included, commonly associated with NARP (neuropathy, ataxia, retinitis pigmentosa) syndrome. This patient presented at age 19 with both myoclonic and tonic‐clonic seizures at a frequency of greater than once a month. An EEG performed at age 38 showed a slow background with generalized epileptiform abnormalities.

#### *Recessive* TRIT1 *Mutation*

One patient homozygous for a *TRIT1* missense mutation was included. This patient developed seizures at age 10 years. He suffered infrequent (<1/month) myoclonic absence and bilateral convulsive seizures. An EEG performed at the age of 23 showed a normal background with focal epileptiform abnormalities over both temporal lobes.

Other genotypes in the cohort in which no epilepsy was found were: *PEO1* (n = 13), *OPA1* (n = 7), m.14709T>C (n = 5), *RRM2B* (n = 4), m.12258A>G (n = 2), succinate dehydrogenase deficiency (n = 2), *SPG7* (n = 2), m.4298G>A, m.7587T>C, m.7989T>C, m.618T>G, m.12283G>A, m.1624C>T, *TYMP*, and *YARS2* (all n = 1).

### Follow‐up

Patients were followed up between their time of inclusion to the cohort (May 1, 2005 to May 1, 2008) and January 1, 2015 through their routine clinic appointments. Total length of follow‐up was 1,245 person‐years (mean, 6.85; range, 1–10); 944 person‐years for patients without epilepsy and 301 person‐years for those with epilepsy. Patients are referred from throughout the UK and Ireland, and 13 cases declined further follow‐up in Newcastle because of the large distance involved (2 cases moved abroad). Three further cases were lost to follow‐up for unknown reasons, and 20 cases died. Nevertheless, our total of 1,245 years of follow‐up represents 87.7% of the 1,419 maximum possible years of follow‐up had all surviving patients remained in the cohort until 2015.

### Risk of Developing Epilepsy

At the time of cohort closure (May 1, 2008), 140 of the 182 patients did not have a history of epilepsy. Three of these patients subsequently developed epilepsy during the period of follow‐up. All carried the m.3243A>G mutation. The mean age at which these individuals had their first seizure was 25 years (range, 20–27), and in each case this was in the context of a stroke‐like episode. These three events arose from a total of 258 years of follow‐up free from epilepsy among the m.3243A>G group, giving an approximate risk of developing epilepsy of 1.2% per year for patients carrying the m.3243A>G mutation. Because no patient carrying another genetic mutation developed epilepsy during the period of follow‐up, it is not possible to calculate the risk of developing epilepsy in these genotypes.

### Risk of Stroke‐Like Episodes

Eleven of 63 patients (17.5%) with the m.3243A>G mutation had a previous history of stroke‐like episodes at the time of cohort closure. In 3 of these patients, seizures occurred exclusively in association with the stroke‐like episodes, these patients remaining seizure free at other times. A further 4 patients (6.3%) with the m.3243A>G mutation had a first episode of stroke‐like episode during the follow‐up period. This included the 3 patients who developed epilepsy during the follow‐up period. In all cases, the stroke‐like episodes were associated with focal seizure activity, which was treated aggressively with maximal antiepileptic medication and intravenous rehydration. These events arose from a total of 344 years of follow‐up free from stroke‐like episode, equating to an approximate risk of developing a first stroke‐like episode of 1.16% per year in patients carrying the m.3243A>G mutation.

The patient with the m.12147A>G mutation had initially presented with a stroke‐like episode as described above. He suffered no further events during the follow‐up period. No stroke‐like episodes occurred in any of the other genotypes included in the study.

### Risk of Status Epilepticus

Status epilepticus occurred in 5 of the patients with m.3243A>G mutation (7.9%). In all cases, this was in the setting of a stroke‐like episode, and in all cases this had occurred before cohort closure in 2008. The patient with m.12147G>A had presented with status epilepticus at age 17 years, but was seizure free at the time of cohort closure. No episodes of status epilepticus were observed in any of the other genotypes included in the study, and no further episodes of status epilepticus occurred during the follow‐up period; consequently, it was not possible to calculate an incidence rate.

### Risk of Death

Twenty of the patients with mitochondrial disease died during the period of follow‐up, giving an overall case fatality rate of 1.61% for all patients with mitochondrial disease. This comprised 5 of the 42 patients with epilepsy and 15 of the 140 patients without epilepsy.

In the 5 patients with epilepsy who died, 2 patients died of respiratory failure, 1 from cardiac failure, 1 from drowning not associate with an epileptic seizure, and in 1 case the cause of death was unknown. In none of these 5 cases was the cause of death thought to be related to an epileptic seizure or to sudden unexplained death in epilepsy. This approximates to a case fatality rate in patients with mitochondrial disease and epilepsy of 1.66%. The mean age of patients with epilepsy was 40.2 years, giving a standardized mortality ratio of 2.96 (95% confidence interval [CI]: 0.96–6.74) compared to the UK control population.

In the 15 patients without epilepsy who died, the commonest cause was pneumonia (n = 3), followed by cardiac failure (n = 2), lung cancer (n = 2), intracerebral hemorrhage (n = 1), and complications of abdominal surgery (n = 1). One patient died of drowning, but there was no suggestion that this had occurred as a result of an epileptic seizure. In 5 patients, the cause of death was unclear. The approximate case fatality rate in patients with mitochondria disease but without epilepsy was 1.60%. Mean age of patients without epilepsy was 50.7 years, giving a standardized mortality ratio of 2.83 (95% CI: 1.59–4.63) compared to the UK control population.

## Discussion

We present the first large‐scale cohort study of epilepsy in adult patients with genetically defined mitochondrial disease. We find an overall prevalence of epilepsy of 23.1% in this cohort. The Newcastle mitochondrial service is one of only three highly specialized referral centers for the whole of the UK and has been reviewing and investigating patients with suspected mitochondrial disease for over two decades. Furthermore, our diagnostic laboratory carries out all genetic testing for mitochondrial disease in the region, and strenuous efforts are made to follow up both affected individuals and their families. We further attempted to obtain a true prevalence figure by following up nonattenders at clinic with telephone interviews. For these reasons, it is unlikely that there are many adult patients with mitochondrial disease who were not included in the cohort. Nevertheless, we cannot rule out bias because of patients with mitochondrial disease being missed from our study population.

We limited our study population to adult patients because despite being a national referral center, we felt that we would not achieve sufficient numbers of pediatric patients to reach meaningful population‐based conclusions. Some pediatric patients with mitochondrial disease present with a severe epilepsy syndrome and often do not survive into adulthood. This survival bias undoubtedly alters the prevalence in adult patients, and, consequently, our reported incidence and prevalence rates specifically relate to patients presenting in adulthood.

Strenuous efforts are made to keep in touch with patients who have been observed within our service, but inevitably patients who are sometimes traveling hundreds of miles to attend outpatient appointments decline further follow‐up. We achieved a follow‐up rate of 87.7% of the maximum possible number of patient years of follow‐up, but accept that our follow‐up data are incomplete. However, we feel that given our close relationship with these patients and their families and our recognition as a national center for the care of these diseases, it is unlikely that a substantial proportion of those lost to follow‐up will have suffered significant epilepsy‐related morbidity or mortality without our knowledge.

Our cohort included a wide range of genetic defects, as well as a wide range of severity from severely affected individuals to oligosymptomatic relatives detected through family screening. Although we find an overall prevalence of epilepsy of 23.1% for the cohort as a whole, there are clear differences between genotypes. In many genotypes, such as m.14709T>C, *OPA1*, and *PEO1*, no patients with epilepsy were found. Prevalence in the commonest genotype (m.3243A>G) was 34.9%, and almost all (92.3%) of the patients with the m.8344A>G point mutation had seizures. Epilepsy was also noted in patients with the m.12147G>A, m.8993T>G, and recessive *TRIT1* mutations. However, only single patients with this genotype were included, and, consequently, there were no other patients carrying the same genotype who did not have epilepsy. The true prevalence in these genotypes therefore cannot be determined, although the implication is that this may be very high. One patient each with single large‐scale deletion and multiple mtDNA deletions of unknown nuclear gene had epilepsy. However, in both cases, the epilepsy had presented at a very young age, many years before the diagnosis of mitochondrial disease was made, and it seems likely that these are secondary to causes other than mitochondrial disease.

Why these differences in epilepsy risk between genotypes should exist is largely unknown. Imaging studies have shown that genotypes with a high prevalence of epilepsy, such as m.3243A>G and m.8344A>G, tend to show more changes in the cerebral cortex than genotypes with a low prevalence of epilepsy, such as large‐scale single deletion.^26^ However, why these mutations are preferentially expressed within the brain is largely unexplained. Within the cortex, cortical interneurons seem particularly vulnerable to mitochondrial dysfunction,^27^ but, again, why these cells are more affected in one genotype versus another remains largely unknown.

A wide range of seizure phenotypes were observed, and, unsurprisingly, individual patients could have several distinct seizure types. Whereas all of the patients with the m.8344A>G and epilepsy exhibited myoclonic jerks, for every other genotype the commonest seizure manifestation was with focal seizures. The m.8344A>G mutation shows a largely homogeneous distribution within the body,^28^ including in neuronal tissues, and it may be for this reason that the commonest seizure type is generalized. In contrast, the m.3243A>G mutation shows large differences in mutation load between and within tissues,^29^ with a particularly high mutation load in cerebral vascular smooth muscle cells.^30^ Energy failure is one plausible mechanism whereby neuronal dysfunction develops in mitochondrial disease,^31^ and it may be that localized perfusion deficits predispose to focal seizures. The extreme expression of this potential perfusion/activity mismatch is the stroke‐like episode.^32^, ^33^ In keeping with other accounts, we find that all of these events are associated with focal seizure activity. In all of the cases, the focal seizure activity was aggressively treated with maximal antiepileptic medication therapy and intravenous rehydration. Although we cannot be certain that this influenced outcome, our practice in these cases is to treat the seizure activity with high doses of intravenous phenytoin or levetiracetam while avoiding agents such as sodium valproate in patients with *POLG* mutations, because this agent may compromise mitochondrial function.^34^ Only 3 patients with other genotypes were taking sodium valproate during the period of follow‐up. In 1 patient with the m.3243A>G mutation, it was effective in suppressing myoclonic jerks. However, in 1 patient with the m.8344A>G mutation, it proved ineffective in controlling myoclonic jerks and was substituted for lamotrigine; and in 1 patient with the m.8993T>G mutation, it proved similarly ineffective. None of these patients suffered any side effects attributable to the medication, suggesting that it is a safe drug to use in patients who do not harbor *POLG* mutations. However, it does not appear to be particularly effective, and given the recent evidence of the efficacy of levetiracetam in the treatment of myoclonus in patients with mitochondrial disease,^35^ we would regard sodium valproate as, at best, a second‐line treatment.

As well as differences in epilepsy prevalence and seizure manifestations, we find significant differences in the risks of epilepsy‐related morbidity. Almost half of the patients with the m.3243A>G mutation had a previous history of stroke‐like episode, and there was a high risk (1.16% per year) of developing stroke‐like episodes in the remainder. Interestingly, we found no stroke‐like events in the patients with the *POLG* mutation. This is in contrast to the high rates reported in other cohorts.^36^, ^37^ We have previously published on this group, the majority of whom presented with a sensory neuronopathy rather than with CNS disease.^38^ However, 3 further patients with the *POLG* mutation presented after 2008 and were consequently not included. All of these patients had seizures and stroke‐like episodes, suggesting that the true prevalence of stroke‐like episodes in this genotype may be higher than our cohort suggests.

Although this is the largest cohort study of patients with mitochondrial disease published so far, the number of deaths, particularly in the epilepsy group, was small. Consequently, the confidence intervals on the standardized mortality ratio values are broad. Nevertheless, the case fatality rates and standardized mortality rates in our cohort are similar to those published in patients with other forms of epilepsy.^39^, ^40^ Interestingly, the standardized mortality rate in our cohort was similar between patients with and without epilepsy. This and the observation that none of the deaths were attributed to epilepsy further suggests that those deaths that did occur were as a result of complications (primarily cardiorespiratory) secondary to mitochondrial disease per se rather than any additional mortality secondary to the presence of seizures.

Our study reports the first prospective study of the prevalence of epilepsy in a large cohort of adult patients with mitochondrial disease. We find a high prevalence of epilepsy in the cohort as a whole, but with striking differences in the prevalence between genotypes. The epilepsy presents at a young age and patients, particularly those carrying the m.3243A>G mutation, remain at high risk of status epilepticus and stroke‐like episodes. This information will be of use in offering prognostic advice to adult patients diagnosed with mitochondrial disease and is a first step in developing more rational treatment strategies for those patients in whom epilepsy develops.

## Authorship

R.G.W., H.D., G.S.G., M.O.C., R.W.T., and D.M.T. were responsible for concept and design of the study.

R.G.W., H.D., Y.N., N.Z.L., R.H., A.M.S., V.N., G.S.G., R.M., R.W.T., and D.M.T. were responsible for data acquisition and analysis. R.G.W., H.D., Y.N., R.H., A.M.S., G.S.G., R.M., R.W.T., D.M.T. were responsible for drafting the text and figures.

## Potential Conflicts of Interest

Nothing to report.

**Table 1:** Prevalence and Seizure Phenotypes in Patients With Mitochondrial Disease and Epilepsy

Genotype	Prevalence of Epilepsy	Age at First Seizure (mean, range)	1 º	2 º	3 º	SLE	SE
Overall	42/182 (23.1%)	29.4 (2–58)					
m.3243A>G	22/63 (34.9%)	32.6 (9–56)	FM (54.5%)	BC (45.5%)	FS (22.7%)	17.5%	7.9%
m.8344A>G	12/13 (92.3%)	23.8 (18‐58)	M (100%)	TC (16.7%)		0%	0%
*POLG1*	3/13 (23.1%)	27.5 (26–33)	M (66.7%)	TC (33.3%)	FDS (33.3%)	0%	0%
Single mtDNA deletion	1/33 (3%)	5 (n/a)	FDS (100%)	BC (100%)		0%	0%
Multiple mtDNA deletions	1/14 (7.1%)	2 (n/a)	FM (100%)	BC (100%)		0%	0%
m.12147A>G	1/1 (100%)	17 (n/a)	FM (100%)	BC (100%)		100%	100%
m.8993T>G	1/1 (100%)	19 (n/a)	M (100%)	TC (100%)		0%	0%
p.(Arg323Gln) *TRIT1*	1/1 (100%)	10 (n/a)	M (100%)	TC (100%)		0%	0%

Estimates of prevalence are taken at the time of cohort closure on May 1, 2008. 1 º = most common seizure type; 2 º = second most common seizure type; 3 º = third most common seizure type.

M = myoclonic; FM = focal motor; FS = focal sensory; FDS = focal dyscognitive; TC = tonic‐clonic; BC = focal seizures evolving to bilateral convulsive seizure; SLE = stroke‐like episode; SE = status epilepticus; n/a = not applicable.

## Supporting information

Additional supporting information can be found in the online version of this article

Supporting Information Table.
