# Supplementary material for: Epilepsy in adults with mitochondrial disease: A cohort study
Source: Ann Neurol. 2015 Nov 17;78(6):949–57. doi: 10.1002/ana.24525 (PMC4737309; doi:10.1002/ana.24525)
Supplement: Supplementary file 1 — Supporting Information Table. [file ANA-78-949-s001.docx]

TABLES

**Supplementary Table 1. Seizure phenotype, frequency and anti-epileptic drug usage in patients with mitochondrial disease and epilepsy.**

| ID | Genotype | Age at first seizure | Age at cohort entry | 1º | 2 º | 3 º | Seizure frequency | Anti-epileptic medications | SLE | SE |
| --- | --- | --- | --- | --- | --- | --- | --- | --- | --- | --- |
| 1 | m.3243A>G | 9 | 16 | M | FS |  | <1/month | CBZ,CLON,LEV | Y | Y |
| 2 | m.3243A>G | 14 | 17 | FM | BC |  | Nil | CBZ,CLOB,LEV | Y | Y |
| 3 | m.3243A>G | 14 | 23 | FDS | FM | BC | >1/week | CLOB,LEV,LTG |  |  |
| 4 | m.3243A>G | 15 | 27 | M | FM |  | >1/day | LEV,LTG,VAL | Y | Y |
| 5 | m.3243A>G | 16 | 21 | FDS |  |  | >1/month | LEV,TOP | Y |  |
| 6 | m.3243A>G | 16 | 22 | TC |  |  | <1/month | LEV |  |  |
| 7 | m.3243A>G | 20 | 24 | FDS |  |  | <1/month | nil | Y |  |
| 8 | m.3243A>G | 20 | 23 | TC |  |  | nil | LTG |  |  |
| 9 | m.3243A>G | 20 | 16 | BC |  |  | <1/month | CLON,LEV,PHE | Y | Y |
| 10 | m.3243A>G | 27 | 19 | BC |  |  | <1/month | CLOB,LEV | Y |  |
| 11 | m.3243A>G | 30 | 32 | FS | FM |  | >1/week | LEV,PHE | Y |  |
| 12 | m.3243A>G | 32 | 30 | M |  |  | <1/month | nil |  |  |
| 13 | m.3243A>G | 33 | 43 | FS | FM | BC | nil | CBZ | Y | Y |
| 14 | m.3243A>G | 35 | 50 | FM | BC |  | <1/month | CBZ | Y |  |
| 15 | m.3243A>G | 38 | 38 | FM | BC |  | >1/week | CBZ,LTG |  |  |
| 16 | m.3243A>G | 42 | 44 | FS | FM |  | nil | CBZ,LEV | Y |  |
| 17 | m.3243A>G | 43 | 45 | FDS |  |  | >1/week | LTG | Y |  |
| 18 | m.3243A>G | 45 | 46 | FM | BC |  | <1/month | LEV,PHE | Y |  |
| 19 | m.3243A>G | 46 | 55 | M | TC |  | >1/week | LEV | Y |  |
| 20 | m.3243A>G | 50 | 57 | FM | BC |  | nil | CBZ |  |  |
| 21 | m.3243A>G | 53 | 57 | FM | BC |  | >1/week | CBZ,LTG | Y |  |
| 22 | m.3243A>G | 56 | 53 | FS | FM |  | <1/month | CBZ |  |  |
| 23 | m.8344A>G | 18 | 33 | M |  |  | >1/day | CLOB,LEV |  |  |
| 24 | m.8344A>G | 20 | 25 | M |  |  | >1/day | CLOB,LEV |  |  |
| 25 | m.8344A>G | 20 | 26 | M |  |  | >1/day | nil |  |  |
| 26 | m.8344A>G | 23 | 22 | M | TC |  | >1/day | LTG |  |  |
| 27 | m.8344A>G | 25 | 58 | M |  |  | >1/day | CLON,GBP,LEV |  |  |
| 28 | m.8344A>G | 25 | 41 | M |  |  | >1/day | LEV |  |  |
| 29 | m.8344A>G | 37 | 50 | M | TC |  | >1/day | CLON,LEV,VAL |  |  |
| 30 | m.8344A>G | 41 | 53 | M |  |  | >1/day | nil |  |  |
| 31 | m.8344A>G | 49 | 55 | M |  |  | >1/day | LEV |  |  |
| 32 | m.8344A>G | 51 | 50 | M |  |  | >1/day | LEV |  |  |
| 33 | m.8344A>G | 54 | 53 | M |  |  | >1/day | LEV |  |  |
| 34 | m.8344A>G | 58 | 58 | M |  |  | >1/day | nil |  |  |
| 35 | *POLG1* | 26 | 50 | M | TC |  | >1/day | CLON,LTG |  |  |
| 36 | *POLG1* | 29 | 39 | M |  |  | nil | nil |  |  |
| 37 | *POLG1* | 33 | 50 | FDS |  |  | <1/month | CBZ |  |  |
| 38 | single mtDNA deletion | 5 | 40 | FDS | BC |  | <1/month | LTG,PHE |  |  |
| 39 | multiple mtDNA deletions | 2 | 53 | FM | BC |  | >1/week | LTG,PHE |  |  |
| 40 | m.12147A>G | 17 | 23 | FM | BC |  | nil | CBZ | Y | Y |
| 41 | m.8993T>G | 19 | 33 | M | TC |  | >1/month | LEV, VAL |  |  |
| 42 | p.(Arg323Gln) *TRIT1* mutation | 10 | 16 | M | TC |  | <1/month | CLON,LEV |  |  |

Supplementary Table 1: 1º = primary seizure type, 2º = secondary seizure type, 3º = tertiary seizure type. Seizure frequency refers to the frequency of the primary seizure type. M = myoclonic, FM = focal motor, FS = focal sensory, FDS = focal dyscognitive, TC = tonic-clonic, BC = focal seizures evolving to bilateral convulsive seizure. CBZ = carbamazepine, CLOB = clobazam, CLON = clonazepam, GBP = gabapentin, LEV = levetiracetam, LTG = lamotrigine, PHE = phenytoin , TOP = topiramate, VAL = valproate. SLE = stroke-like episode, SE = status epilepticus.
